# An efficient *in vitro* organogenesis protocol for the endangered relic tree species *Bretschneidera sinensis* and genetic fidelity assessment using DNA markers

**DOI:** 10.3389/fpls.2024.1259925

**Published:** 2024-04-10

**Authors:** Xuetong Yan, Keyuan Zheng, Peng Li, Xin Zhong, Zongwei Zhu, Huijing Zhou, Mulan Zhu

**Affiliations:** ^1^ Shanghai Key Laboratory of Plant Functional Genomics and Resources, Shanghai Chenshan Botanical Garden, Shanghai, China; ^2^ National Key Laboratory of Plant Molecular Genetics (NKLPMG), Chinese Academy of Sciences (CAS) Center for Excellence in Molecular Plant Sciences, Shanghai, China; ^3^ College of Forestry, Fujian Agriculture and Forestry University, Fuzhou, China

**Keywords:** *Bretschneidera sinensis*, mature zygotic embryo, organogenesis, genetic fidelity, molecular markers

## Abstract

*Bretschneidera sinensis* is a monotypic species of rare and tertiary relic trees mainly distributed in China. *B. sinensis* is a potentially valuable horticultural plant, which has significant ornamental and research value, and is a crucial tool for the study of phylogeography. The artificial cultivation of *B. sinensis* is of great scientific value and practical significance. In this study, we developed a direct organogenesis process of *B. sinensis* using mature zygotic embryos as initial materials. The highest sterile germination induction (54.5%) from the mature zygotic embryo was obtained in a Murashige and Skoog (MS) medium with 2.0 mg·L^−1^ 6-benzylaminopurine (6-BA) and 0.2 mg·L^−1^ α-naphthaleneacetic acid (NAA). The highest percentage of shoot regeneration (90.37%) was attained using 1.0 mg·L^−1^ 6-BA and 0.01 mg·L^−1^ NAA in the MS medium. The Woody Plant Medium (WPM) had the greatest adventitious shoot elongation rate of 93.33%. The most optimized rooting rate was 88.89% in a half-strength MS medium containing 2.0 mg·L^−1^ indole-3-butyric acid (IBA) and 1.0 mg·L^−1^ NAA. The genetic fidelity of *in vitro* regenerated plantlets was assessed using inter-simple sequence repeats and random amplified polymorphic DNA molecular markers, confirming the genetic uniformity and stability of regenerated *B. sinensis* plantlets. Our research presents an effective *in vitro* propagation system for *B. sinensis*, laying the groundwork for its germplasm conservation and large-scale production while maintaining high genetic integrity.

## Introduction

*Bretschneidera sinensis* (Hemsl) (NCBI: txid28529; 2n = 18), also known as the Bole tree, is a valuable tree species of the Akaniaceae (turnipwood) family (Montaut et al., 2015; Wang et al., 2018). Fossil evidence shows a wide distribution of *B. sinensis* in the Northern Hemisphere during the Late Miocene, which declined over geological time and was restricted to a narrow geographical range in the distant mountainous areas of southern China, with some dispersed individuals appearing in eastern Asian countries in modern times (Romero and Hickey, 1976). Almost all relict species in the wild are endangered and currently restricted to historical refugia (Isah, 2015). *B. sinensis* is tall and straight and has attractive white flowers with pink or reddish veins, making it a potential horticultural species, according to Liu et al. (2022). Its leaf appendages have a unique structure, the upper epidermis is covered with striated cuticles, the lower epidermis is covered with flower-like papillae, and the stomatal apparatus is found only on the lower epidermis. The characteristics of *B. sinensis* leaf appendages reflect its long-term adaptation to its environment (Tu et al., 2012), and the species also has medicinal value for arthralgia and myalgia (Liu et al., 2010). *B. sinensis* is a model plant for research on angiosperm phylogeny, paleogeography, and paleoclimatology, making it an important phylogeographic research tool (Liu et al., 2021).

*B. sinensis* is a plant species with an extremely small population, which creates an increasing need to improve conservation techniques for its preservation (Yang et al., 2020). Effective conservation efforts are essential to prevent numerous species from extinction owing to various biotic and abiotic causes leading to their endangered status (Tommasi and Scaramuzzi, 2004). *B. sinensis* often grows beneath broadleaved forests, and its distribution range is constrained and confined. Its growth is very slow, and the juvenile phase is long (Liu et al., 2022). It is a mycorrhizal species, and the mortality rate of annual seedlings is high in the absence of fungal development, while the natural germination rate is less than 8% in the wild (Guo et al., 2007). This gap between seed dispersal and seedling emergence results in substantial losses, exacerbated by long-term ecological environment damage, which leads to reduced fruiting and difficulties in natural renewal, and ultimately contributes to the endangered status of *B. sinensis*. The International Union for Conservation of Nature (IUCN) has listed *B. sinensis* as “Endangered” on the IUCN Red List. *B. sinensis* is also recognized as a Category I endangered species in China’s Key List for the Protection of Wild Plants (Zhang et al., 2022), which requires effective conservation measures. Its survival and dissemination depend on ensuring germination and seedling growth at suitable times and locations (Qi et al., 2009; Chen et al., 2020). Thus, *ex situ* conservation must be performed to ensure survival (Qiao et al., 2012). Effective conservation can be achieved by the application of plant tissue culture through *in vitro* regeneration of the species. *In vitro* regeneration technology has many advantages, such as high production efficiency and the ability to obtain a large number of plant seedlings within a short period of time, greatly reducing the breeding cycle. The process is stable, reliable, and controllable because it is not affected by external environmental factors. *In vitro* regeneration has been widely used to protect numerous endangered monogenous woody species, such as *Ginkgo biloba* (Tommasi and Scaramuzzi, 2004), *Glyptostrobus pensilis* (Yuan et al., 2023), *Metasequoia glyptostroboides* (Xiong et al., 2020), and *Cercidiphyllum japonicum* (Fu et al., 2012). However, a few studies have been conducted on *B. sinensis* shoot regeneration. In experiments where shoots from *B. sinensis* seedlings were used as explants, researchers found a very low regeneration efficiency, with only 3.2 regenerated plantlets per explant, and the entire protocol took a long time (Guo et al., 2007). At present, there is no efficient tissue culture method for *B. sinensis*. Therefore, species-specific *in vitro* regeneration systems for *B. sinensis* whole-plant regeneration need to be established and optimized.

Evaluating the genetic uniformity of shoots using plant tissue culture is important owing to somaclonal variations in *in vitro* regenerated plantlets. DNA molecular marker analysis has been used to identify intraspecific variations in plant populations (Pillai et al., 2012). Presently, it can also be used to examine somaclonal variations in *in vitro* propagated plantlets. Start codon targeted polymorphism (Erişen et al., 2020), inter-simple sequence repeat (ISSR), random amplified polymorphic DNA (RAPD) (Ahmed et al., 2017; Kadapatti and Murthy, 2021), simple sequence repeats (Ioannidis et al., 2022), and amplified fragment length polymorphism (Mehta et al., 2011) have been leveraged to assess the genetic stability of regeneration plantlets. ISSR and RAPD have garnered the greatest support because they are frequently employed to investigate the genetic fidelity of regenerated species. However, research into the direct organogenesis regeneration protocol using limited *B. sinensis* explants is still lacking. Similarly, studies assessing the genetic fidelity of tissue-cultured plantlets—especially those transferred over 10 times—using molecular markers on initially transferred explants remain scarce.

In the present study, we aimed to establish a conservation method through direct adventitious shoot organogenesis process of *B. sinensis* using mature zygotic embryos as initial materials. The application of this advanced *in vitro* regeneration technology can be employed for endangered plant species *ex situ* conservation. It can also facilitate the supply of plantation seedlings, which offers a basis for boosting the proliferation of *B. sinensis* as a new landscape tree resource. This study represents, to our knowledge, the inaugural report on a comprehensive and efficacious regeneration system that illustrates how to obtain regenerated plantlets from limited explants in *B. sinensis*. In addition, we analyzed the genetic fidelity of regenerated *B. sinensis* shoots for the first time. This method not only paves the way for the ex situ conservation of endangered species but also contributes to ecological restoration efforts and biodiversity conservation by enabling the proliferation of *B. sinensis*.

## Materials and methods

### Seed preparation

**Figure 1:**
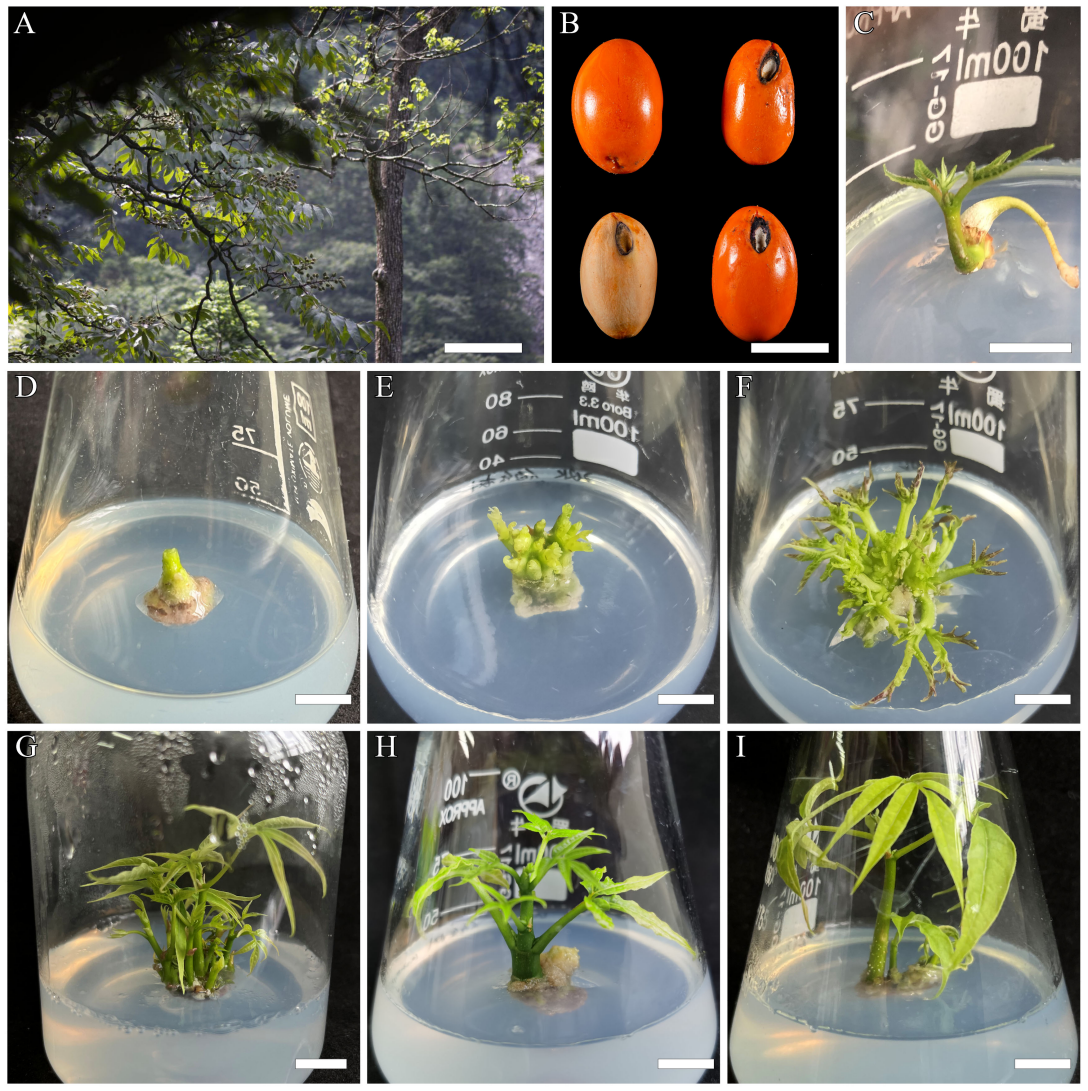
*In vitro* shoot induction and plantlets regeneration process of *B*. *sinensis* from mature zygotic embryos. **(A)** Adult tree of *B*. *sinensis*, bar = 33 cm. **(B)** Seed and fruits of *B*. *sinensis*, the bottom left is the seed with the flesh removed, bar = 1 cm. **(C)** Zygotic embryo germination, bar = 1.2 cm. **(D)** Initiation of adventitious shoots, bar = 0.9 cm. **(E, F)** Multiple shoots induction, bar = 0.9 cm. **(G–I)** Regenerated plantlets with well-developed elongation, bar in **(G)** = 1.12 cm, bar in **(H, I)** = 0.9 cm.

Thirty-three *B. sinensis* seeds randomly selected from four thriving mature plants from the BeiGang Forest Farm, county Jing’an, Yichun (114°57′32.5″E, 28°55′11.38″N) of Jiangxi province, China, were provided by Chenshan Herbarium (**Figures 1A, B**). The fruit pulp was removed and seeds were rinsed under tap water and distilled water, then the water was blotted with filter paper. Surface sterilization was performed with 1‰ [v/v] potassium permanganate for 3 min, 70% [v/v] ethanol for 1 min, and then 20% [v/v] sodium hypochlorite for 12 min; sterilization procedure involved rinsing three to five times using sterile water. Afterwards, the sterilized *B. sinensis* seeds were placed in a pre-culture Murashige and Skoog (1962) (MS) mineral solution medium with 1‰ [v/v] plant preservative mixture (Yeasen, Shanghai, China) and without plant growth regulators (PGRs). All plant media for this study contained MS vitamins and were augmented with sucrose (3%) and gelled with agar (5.0 g·L^−1^). The pH was adjusted to 6.0 before adding agar and autoclaving for approximately 20 min at 121°C. The *B. sinensis* seeds in cultures were stored in a 16/8-h cycle of light and darkness at 25 ± 2°C, and maintained at 70% relative humidity. After 3 days, the *B. sinensis* seeds were checked for sterility, the endosperm was removed with a scalpel, and zygotic embryos were used for the subsequent experiments.

### Germination from sterile zygotic embryos

The zygotic embryos were obtained in sterile environments and were used for inducing the zygotic embryos germination. Limited by the low fruiting rate of *B. sinensis*, 33 zygotic embryos were transferred in germination induction medium (GIM) in a 100-mL Erlenmeyer flask. The GIM media for this study contained MS mineral solution and vitamins, and were augmented with sucrose (3%) and gelled with agar (5.0 g·L^−1^). MS medium with two concentrated 6-benzylaminopurine (6-BA) (0.2 mg·L^−1^ and 2 mg·L^−1^) combined with the same amount of α-naphthaleneacetic acid (NAA) was used as the GIM for explant germination, each group had 11 zygotic embryos for the experiment, and the pH of the medium was adjusted to 6.0. With cool white fluorescent light of 60 μmol·m^−2^·s^−1^, the zygotic embryo germination was sustained in a 16/8-h cycle of light and darkness by maintaining the temperature at 25 ± 1°C and the relative humidity at 70% (**Figure 1C**). The germination induction percentage was recorded after 2 weeks (**Table 1**).

**Table 1:** The germination and callus induction rate of *B. sinensis* zygotic embryos.

Concentrations of plant growth regulators (mg·L^−1^)		Zygotic embryos	
6-BA	NAA	Germination rate (%)	Callus induction rate (%)
2.0	0.2	54.5	0
2.0	2.0	36.3	9.1
0.2	2.0	27.2	27.2

### Proliferation of Shoots

The cotyledons and radicles of germinated zygotic embryos were excised and the remaining cotyledons, accounting for approximately 1–1.5 cm, were transferred to the same GIM medium until enough adventitious shoots were obtained. These shoots were used for the subsequent proliferation experiments. Afterwards, the adventitious shoots were transferred to solid MS medium added cytokinin 6-BA (0.1–3.0 mg·L^−1^) in combination with auxin NAA at various concentrations (0.01–1.5 mg·L^−1^) for shoot proliferation. The medium for proliferation was augmented with sucrose (3%), gelled with agar (5.0 g·L^−1^), and its pH was adjusted to 6.0 (**Table 2**). In a 100-mL Erlenmeyer flask, each treatment included one to three explants for the regeneration of adventitious shoots with a total of 45 replications for this experiment. With cool white fluorescent light of 60 μmol·m^−2^·s^−1^, keeping an environment temperature at 25 ± 1°C and 70% relative humidity were used to sustain the shoot proliferation cultures in a 16/8-h cycle of light and darkness. The regenerated *B. sinensis* shoots (**Figure 1D**) were sub-cultured using fresh medium (**Figures 1E–G**) every 2 weeks. After 8 weeks, the average shoot number and the adventitious shoot regeneration rate were counted. Subsequently, newly grown *B. sinensis* shoots were cut down and transferred to the shoots’ elongation medium (**Figures 1H, I**).

**Table 2:** Effects of PGRs combination and intensity on adventitious shoot induction.

Order	Concentrations of plant growth regulators (mg·L^−1^)		Shoot regeneration rate (%)	Number of adventitious shoots per explant
	6-BA	NAA		
1	0.1	0.01	52.22 ± 1.1^def^	2.13 ± 0.04^k^
2	0.1	0.03	60.0 ± 10.2^bcde^	3.07 ± 0.04^j^
3	0.1	0.05	50.37 ± 1.96^def^	2.11 ± 0.06^k^
4	0.3	0.03	66.67 ± 3.85^abcde^	5.24 ± 0.14^gh^
5	0.3	0.1	31.1 ± 4.44^fg^	6.11 ± 0.15^fg^
6	0.3	0.15	48.9 ± 4.44^ef^	2.20 ± 0.12^jk^
7	1.0	0.05	80.0 ± 38.5^abc^	8.07 ± 0.20^d^
8	1.0	0.1	90.37 ± 1.96^a^	15.89 ± 0.29^a^
9	1.0	0.3	84.44 ± 5.88^ab^	7.09 ± 0.10^e^
10	1.0	0.5	57.78 ± 5.88^cde^	6.16 ± 0.18^f^
11	2.0	0.1	68.89 ± 0.08^abcde^	11.13 ± 0.24^c^
12	2.0	0.2	75.56 ± 2.22^abcd^	14.49 ± 0.26^b^
13	2.0	0.6	56.3 ± 8.54^cdef^	5.02 ± 0.14^hi^
14	2.0	1.0	31.11 ± 2.2^fg^	4.89 ± 0.20^hi^
15	3.0	0.3	52.08 ± 2.84^def^	8.91 ± 0.18^d^
16	3.0	0.9	75.56 ± 2.22^abcd^	4.29 ± 0.19^j^
17	3.0	1.5	20.0 ± 3.849^g^	2.22 ± 0.04^jk^

Same letters show no significant difference according to Tukey’s multiple comparison test and p ≤ 0.05.

### Elongation of the adventitious shoots

**Figure 2:**
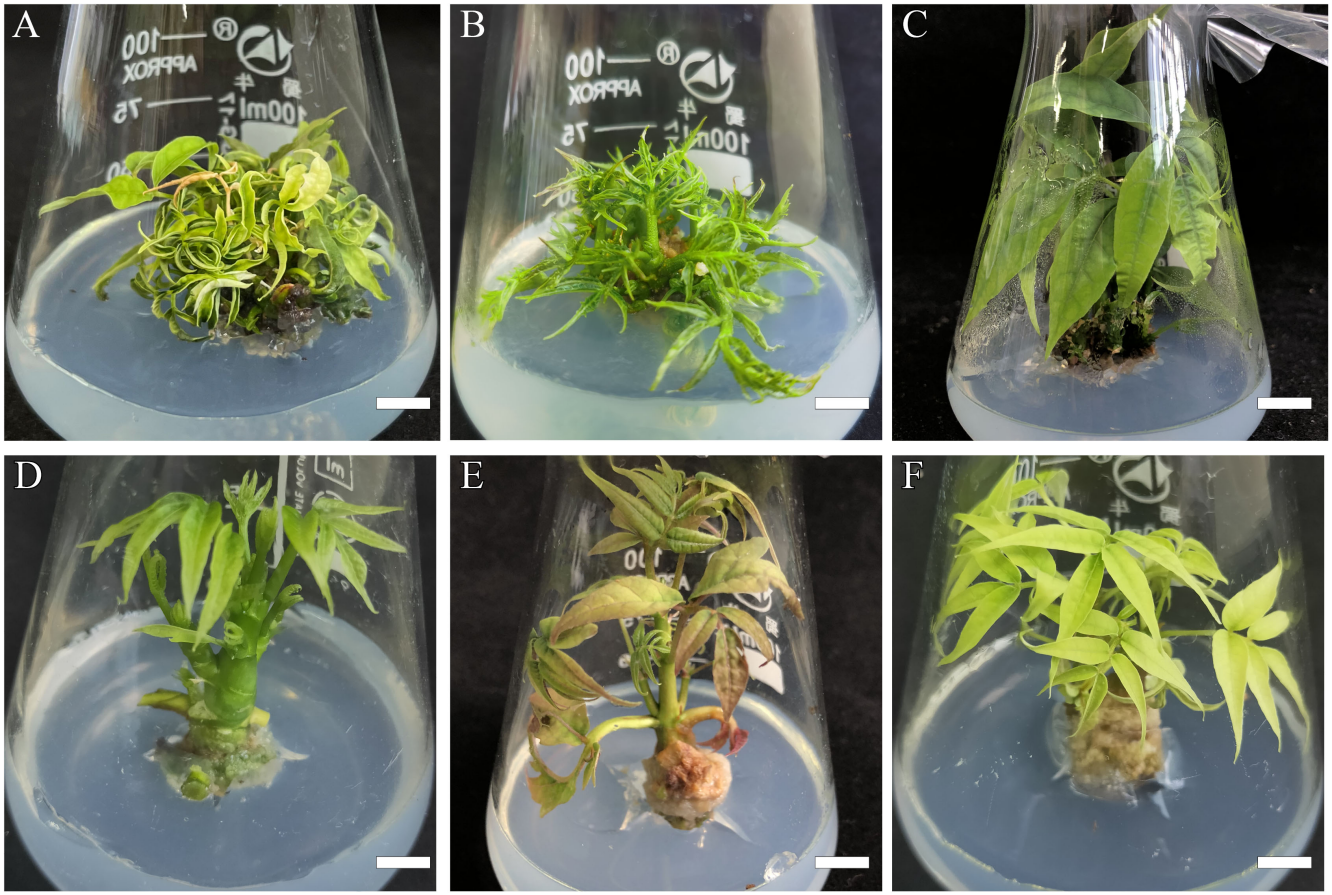
Effects of basic mediums on *B*. *sinensis* elongation and growth conditions. **(A)** Regenerated plantlets in MS basic medium, bar = 0.9 cm. **(B)** Regenerated plantlets in 1/2MS basic medium, bar = 0.9 cm. **(C, D)** Regenerated plantlets in WPM basic medium, bar in C = 0.9 cm, bar in **(D)** = 1.2 cm. **(E)** Regenerated plantlets in 2× DCR medium, bar = 1.2 cm. **(F)** Regenerated plantlets in DCR basic medium, bar = 0.9 cm.

For the *in vitro* shoots’ elongation, adventitious shoots were individually dispersed and transferred to different types of basic media [MS, half-strength (1/2) MS, Woody Plant medium (WPM) (Lloyd Gregory, 1980), Douglas fir cotyledon revised medium (DCR) medium (Gupta and Durzan, 1985), double-strength (2×) DCR]. Each medium was treated with PGRs combined with 1 mg·L^−1^ cytokinins BA and 0.1 mg·L^−1^ auxin NAA, and gelled with agar (5.0 g·L^−1^) and the pH of the medium was adjusted to 6.0 (**Table 3**). Each regenerated *B. sinensis* shoot was transferred to a 100-mL flask, with 45 replications. The shoot elongation cultures in a 16/8-h light/dark cycle were sustained using a cool white fluorescent light of 60 μmol·m^−2^·s^−1^ at an environment temperature of 25 ± 1°C and a relative humidity of 70%. At 3-week intervals, as the *B. sinensis* shoots elongated, they were subcultured using new media with identical ingredients (**Figure 2**). After 6 weeks, the shoot elongation percentage (%) was calculated, and the elongation lengths were measured. Thereafter, regenerated *B. sinensis* shoots were moved to the media for rooting.

**Table 3:** Effects of different basic mediums on *B sinensis* adventitious shoot elongation.

Basic medium	Shoot elongation (%)	Average length (cm)	Description
MS	17.78 ± 4.44^c^	1.97 ± 0.12^c^	Small shoots with crinkle leaves
1/2MS	42.22 ± 5.88^b^	3.13 ± 0.15^b^	Crinkle leaves
WPM	93.33 ± 3.76^a^	5.13 ± 0.07^a^	Healthy green leaves
DCR	71.33 ± 4.33^a^	4.97 ± 0.03^a^	Leaves chlorosis
2DCR	82 ± 5.86^a^	5.03 ± 0.07^a^	Leaves chlorosis

Same letters are not significantly different by Tukey’s multiple comparison test and p ≤ 0.05.

### Rooting of elongated shoots

**Figure 3:**
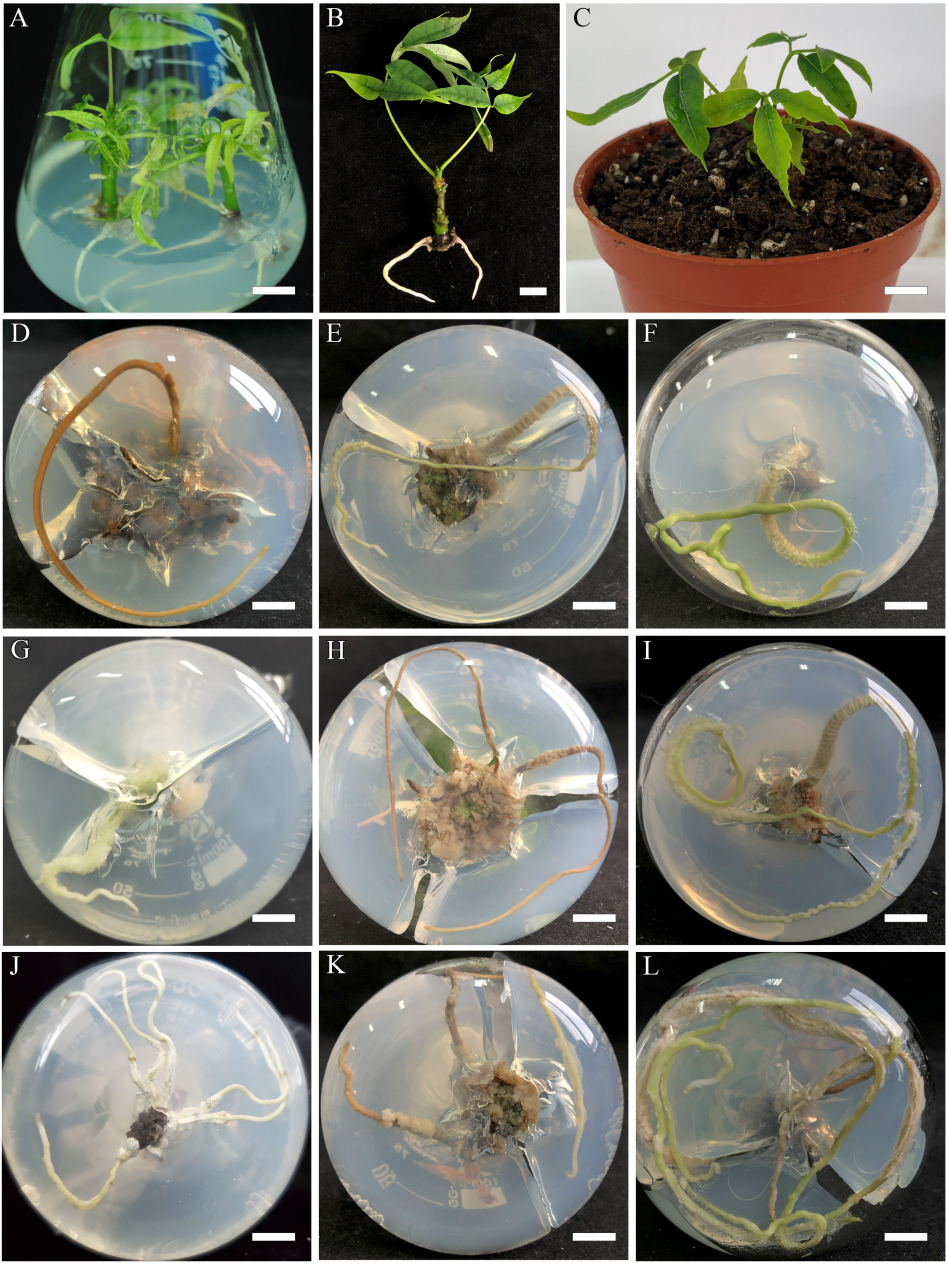
Rooting of *B*. *sinensis* regenerated shoots. **(A)** Rooting of *B*. *sinensis* regenerated shoots, bar = 0.83 cm. **(B)** Regeneration of *B*. *sinensis* plantlets, bar = 0.6 cm. **(C)** Acclimatized plants in 3 weeks, bar = 2 cm. **(D)** 1/2MS (sucrose-), bar = 1.18 cm. **(E)** 1/2MS + IBA 1 mg·L^−1^ + NAA 2 mg·L^−1^, bar = 0.83 cm. **(F)** 1/2MS + 0.5 mg·L^−1^ NAA + 1 mg·L^−1^ IBA, bar = 0.83 cm. **(G)** 1/2MS + 1 mg·L^−1^ NAA + 3 mg·L^−1^ IBA, bar = 0.83 cm. **(H)** 1/2MS+ 1 mg·L^−1^ NAA + 2 mg·L^−1^ IBA (sucrose-), bar = 0.83 cm. **(I)** 1/2MS + 1 mg·L^−1^ NAA + 2 mg·L^−1^ IBA, bar = 0.83 cm. **(J)** 1/2MS + 2 mg·L^−1^ IBA, bar = 0.83 cm. **(K)** 1/2MS + 2 mg·L^−1^ NAA + 1 mg·L^−1^ IBA, bar = 0.83 cm. **(L)** 1/2MS + 2 mg·L^−1^ NAA + 3 mg·L^−1^ IBA, bar = 0.83 cm.

For *B. sinensis* rooting, the elongated shoots (approximately 3–5 cm) were cut and cultured in the 1/2MS and WPM basic media (50 mL) of various strengths with auxin NAA singly or with auxin indole-3-butyric acid (IBA) in polystyrene culture vessels (125 × 110 mm). The media in the experiments all contained 30 g·L^−1^ sucrose, and only in the rooting experiments did some of the media reduce the sucrose content to 20 g·L^−1^, which is labeled in **Table 4**, gelled with agar (5.0 g·L^−1^), and the medium was adjusted to 6.0. For this experiment, there was one regenerating shoot with 45 replications. The rooting cultures in a 16/8-h cycle of light and darkness were sustained using a cool white fluorescent light of 60 μmol·m^−2^·s^−1^, and by maintaining the environment temperature of 25 ± 1°C and relative humidity of 70%. After cultivating for 10 weeks with no subculture, the rooting rate for each treatment was evaluated (**Figure 3**).

**Table 4:** Effect of IBA, NAA, and sucrose on rooting of *B. sinensis* in 1/2 MS and WPM media.

Medium	Concentrations of PGRs (mg·L^−1^)		Concentrations of sucrose (g·L^−1^)	Rooting rate (%)	Description
	IBA	NAA			
1/2 MS			20	20 ± 3.8^g^	Slender
	1.0		20	51.11 ± 4.4^cdef^	Slender and white
	1.0	0.5		46.67 ± 3.8^def^	Thick and green
	2.0	0.5		55.56 ± 5.9^bcde^	Slender
	2.0	1.0		80 ± 3.8^a^	Thick, loose, and white
	2.0	1.0	20	88.89 ± 2.2^a^	Slender, white, robust
	3.0	1.0		68.89 ± 4.4^abcd^	Thick, loose, and white
	2.0			71.11 ± 5.9^abc^	Slender, loose, and white
	1.0	2.0		77.78 ± 2.2^ab^	Thick, loose, and white
	2.0	2.0		73.33 ± 3.8^abc^	Thick, loose, and green
	3.0	2.0		66.67 ± 6.7^abcd^	Thick, loose, and green
WPM	1.0			31.11 ± 2.2^fg^	Slender and white
	1.0	0.5		33.33 ± 3.8^efg^	Slender and white
	2.0	1.0		68.89 ± 2.2^abcd^	Thick, loose and white
	2.0	1.0	20	53.33 ± 3.8^cdef^	Thick, loose and green
	3.0	2.0		35.56 ± 5.9^efg^	Thick, loose and green

Same letters indicate no significant difference according to Tukey’s multiple comparison test and p ≤ 0.05; Sucrose: sucrose content was indicated to be 20 g·L^−1^ (the default sucrose content was 30 g·L^−1^ for the other media).

### Acclimatization

After rinsing the *B. sinensis* plantlets thoroughly under running water, the medium around the roots was washed; then, the plants were transplanted into a plastic container with a 15-cm diameter (**Figure 3C**); the substrate composition contained peat, organic cultivation soil, and perlite at a ratio of 3:6:1 (v/v). Transparent plastic bags were used to cover the plastic pots to preserve humidity under acclimatization at a 16/8-h light/dark cycle; the growth chambers were operated under a cool white fluorescent light of 33.73 μmol·m^−2^·s^−1^ and the environment temperature was maintained at 25 ± 1°C. After 3 weeks, as the plantlets adapted, the plastic covers were progressively taken off. Plant survival rates were assessed 6 weeks later.

### Genetic fidelity analysis

**Figure 4:**
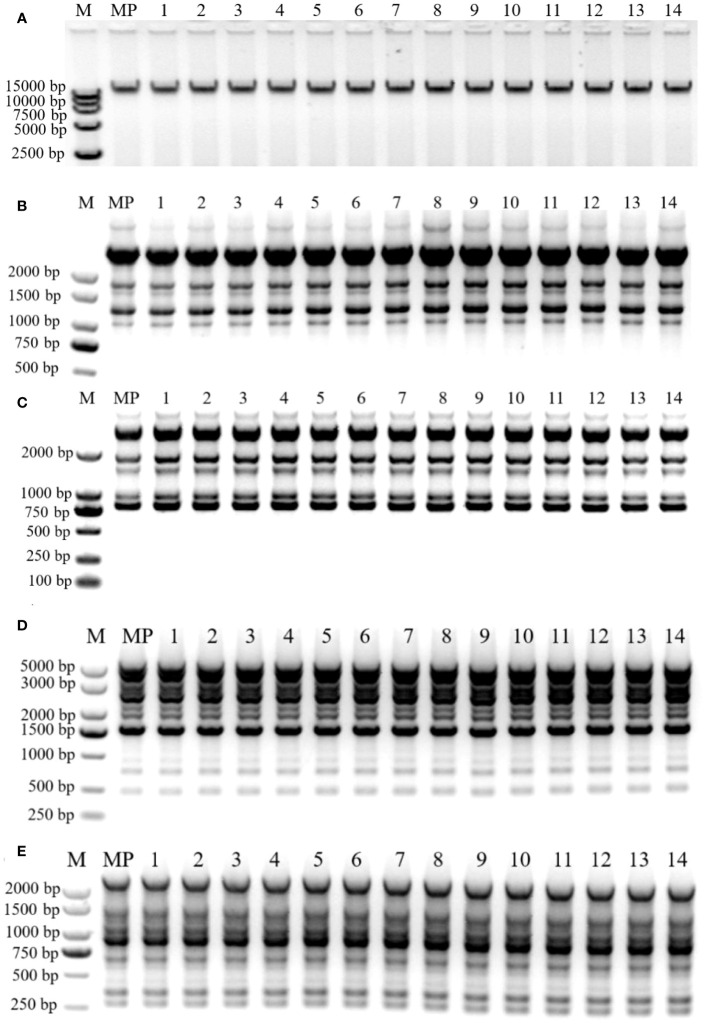
Genetic fidelity of regenerated plants. **(A)** Genomic DNA extraction electrophoresis assay of *B*. *sinensis.* **(B, C)** ISSR profiles generated by PCR amplification with primer UBC-874 **(B)** and UB-C892 **(C)**. Lane M: Molecular marker (100 bp–5 Kb); MP: Mother plant, the first-time transferred plantlet; Lanes 1–14: Regenerated plants transferred over 10 times. **(D, E)** RAPD profiles generated by PCR amplification with primer S130 **(D)** and S144 **(E)**. Lane M: Molecular marker (100 bp–5 Kb for S130; 100 bp–2 Kb for S144); MP: Mother plant, the first-time transferred plantlet; Lanes 1–14: Regenerated plants transferred over 10 times.

One zygotic embryo germinated in medium with cytokinin 2 mg·L^−1^ 6-BA and 0.2 mg·L^−1^ auxin NAA (**Table 1**) was selected, and the experiments were conducted with the seedling line it produced for genetic fidelity analysis. Plantlets transferred for the first time after germination were identified as the mother plant, and plantlets transferred 10 times or more were selected for experiments. Leaves from *in vitro* plantlets that were transferred the first time and over 10 times were collected for DNA extraction. Liquid nitrogen was used to freeze fresh leaves, and the cetyl-trimethyl ammonium bromide (CTAB) technique was used to isolate genomic DNA (Gawel and Jarret, 1991). Agarose gel electrophoresis (0.8%) was used to qualify the purity of the isolated DNA, after the extracted DNA had been added with 7 µL of RNase (100 mg·mL^−1^) and measured using NanoDropC (Thermo Fisher Scientific, Waltham, USA) at 260 nm and 280 nm (**Figure 4A**). The ISSR assay was conducted in 20 μL of reaction mixture containing 10 μL of 2×Taq PCR Mix, 0.5 mmol primers (Sangon, Shanghai, China), and 100 ng of genomic DNA. The ISSR amplification was performed in thermal cycles (Eppendorf ESP-S). The thermal conditions used for the ISSR assay were as follows: initial denaturation at 95°C for 5 min, 40 cycles at 95 for 30, 46°C for 30 s, 72°C for 130 s, and final extension for 10 min at 72°C. The RAPD amplification thermal conditions used in the study were initial denaturation at 95°C for 5 min, 40 cycles at 95°C for 30 s, 38°C for 30 s, 72°C for 130 s, and a final extension for 10 min at 72°C. Amplified products and GelRed staining were resolved in 1.2% agarose gel (1×TAE); then, the gel was documented using a gel doc system (Bio-Rad, Hercules, CA, USA) under UV light. The clear DNA bands were considered when estimating the size of the amplification products using the DL2000 DNA Ladder or the 5,000-bp DNA marker (Vazyme, Nanjing, China). Then, 29 ISSR primers and 20 RAPD primers were evaluated, producing scoreable bands, and repeatable bands were chosen for further amplification.

### Statistical analysis

The following equations were employed to determine various *in vitro* regeneration parameters:


$$\text{Germination rate }\left(\%\right)=\frac{\text{the number of germinated zygotic embryos}}{\text{Initial number of zygotic embryos }}\times 100\%;$$

$$\text{Callus induction rate }\left(\%\right)\;=\frac{\text{the number of callus induced explants}}{\text{the number of initial explants }}\;\times \;100\%;$$

$$\begin{array}{c} \text{ Adventitious shoot induction rate }\left(\%\right)=\\ \frac{\text{the number of explants with adventious shoots}}{\text{the number of initial explants}}\;\times \;100\%; \end{array}$$

$$\begin{array}{c} \text{Adventitious shoot elongation rate }\left(\%\right)=\\ \frac{\text{the number of elongated shoots }}{\text{the number of shoot on the elongation medium}}\;\times \;100\%; \end{array}$$

$$\text{Average shoot length}=\frac{\text{the length of the shoots }}{\text{Initial number shoots for elongation induction}};$$

$$\text{Root induction rate }\left(\%\right)=\frac{\text{the number of the rooted plantlets}}{\text{Initial number of the shoots}}\;\times \;100\%;$$

All experimental results were evaluated using the one-way ANOVA with Tukey’s post-hoc multiple comparison testing except seed germination owing to the insufficient number of explants using SPSS (IBM SPSS Statistics 27.0). At *p* ≤ 0.05, means were deemed statistically significant.

## Results

### Seed preparation and germination

After surface sterilization, 17 of the 33 *B. sinensis* seeds responded, while the others were contaminated and debilitated. With the seed preparation and germination, we found that the highest germination rate (54.5%) was achieved in medium supplemented with 2 mg·L^−1^ 6-BA and 0.2 mg·L^−1^ NAA (**Figure 1C**), followed by 36.3% in the medium with equal amounts of 2 mg·L^−1^ 6-BA and 2 mg·L^−1^ NAA. The germination rate was 27.2% in the medium containing 0.2 mg·L^−1^ 6-BA and 2 mg·L^−1^ NAA, which had the same rate as that of callus induction (**Table 1**).

### Shoot induction and proliferation

For the induction and proliferation of the *B. sinensis* adventitious shoot, when the ratio of cytokinins 6-BA/auxin NAA was 10/1 in the MS media, and the concentration of the two PGRs were 1.0 and 0.1 mg·L^−1^, respectively, 90.37 ± 1.96% of explants regenerated new shoots. Additionally, 16 ± 0.29 shoots formed per explant, which was the highest induction effect. This was followed by the medium with the same concentration of 6-BA and an increased NAA (0.3 mg·L^−1^), which had a shoot regeneration of 84.44 ± 5.88% and a low shoot number per explant of 7 ± 0.10 (**Table 2**). Therefore, the shoot regeneration rate and adventitious shoot number per explant induced by the different formulations were inconsistent and disproportionate (**Table 2**). Both shoot regeneration rates were high in a medium containing 6-BA (1 mg·L^−1^) combined with NAA (0.05 to 0.3 mg·L^−1^). Culture media enriched with 6-BA and NAA at a ratio of 10/1 or lower generally had more adventitious shoots on average.

However, there were certain exceptions. Regardless of whether the ratio of cytokinin 6-BA and auxin NAA is 10/1 or lower than 10/1, a certain concentration must be achieved for some effect. In media containing 0.1 mg·L^−1^ 6-BA and 0.01 mg·L^−1^ NAA, as well as 0.3 mg·L^−1^ 6-BA and 0.03 mg·L^−1^ NAA, both the induction rate and number of adventitious shoots were very low. In addition, when the ratio of 6-BA and auxin was 2/1–3/1, the average number of adventitious shoots in these media was very low, no more than six, and no lower than two (**Table 2**), which is unfavorable for the proliferation of adventitious shoots.

### Effects of the basic medium on elongation and growth status

Before elongation induction, the plantlets were sub-cultured in a fresh shoot regeneration medium for 4 weeks. We used the cytokinin BA/auxin NAA combination with 1/0.1 mg·L^−1^ in five types of different basic media (MS, 1/2MS, WPM, 2×DCR, and DCR) with different nutrient levels for adventitious shoot elongation. The WPM basic medium yielded the optimal elongation rate of 93.33 ± 3.76% and the longest average shoot length was 5.13 ± 0.07 cm (**Figures 2C, D**). This was followed by the 2× DCR and DCR media, with shoot elongation percentages of 82 ± 5.86 and 71.33 ± 4.33%, respectively (**Figure 2E**), and average shoot lengths of 5.03 ± 0.07 cm and 4.97 ± 0.03 cm, respectively (**Figure 2F**), close to the average length of the WPM group. From a growth perspective, plants in the WPM group were green and robust, whereas plants in the 2× DCR and DCR groups exhibited leaf chlorosis.

In the same 1/2MS and MS media supplements, both the shoot elongation induction effect and elongated length were significantly lower (*p*< 0.05) than those in WPM (**Table 3**; **Figures 2A, B**). For the 1/2MS basic medium, 42.22 ± 5.88% of the shoots experienced elongation with an average elongation length of 3.13 ± 0.15 cm. For the MS group, the shoot elongation rates and lengths were considerably lower, at 17.78 ± 4.44% and 1.97 ± 0.12 cm, respectively. Small shoots with crinkle leaves were seen in the MS and 1/2MS groups, and the degree of crinkled leaves in the 1/2MS medium was lower than that in the MS medium, indicating that a suitable basic medium can favor leaf stretching and plantlet elongation. The basic medium considerably boosted both the shoot elongation average and percentage. Thus, the WPM basic medium is relatively suitable for adventitious shoot elongation in *B. sinensis.*

### Rooting

Here, 1/2 MS medium and WPM were used for rooting induction in the basic medium, achieving good rooting (**Figures 3A, B**). The highest adventitious root induction rate was 88.89 ± 2.2% (**Table 4**; **Figure 3H**) in 1/2MS medium supplemented with 2.0 mg·L^−1^ IBA, 1.0 mg·L^−1^ NAA, and sucrose 20 g·L^−1^ (sucrose-), followed by the same component with an additional 10 g·L^−1^ sucrose, with an adventitious root induction rate of 80 ± 3.8% (**Figure 3I**). However, in the WPM medium with the same PGRs, the rate was 68.89 ± 2.2%, the highest adventitious root induction rate in the WPM group.

Compared to the other media, the adventitious root induction rates of the two treatments in the 1/2 MS medium were considerably greater (*p* ≤ 0.05). The combination of NAA and IBA was more effective than auxin alone for rooting. In each basic medium group, the adventitious root induction rate peaked at auxin levels of 3 mg·L^−1^ overall (**Figures 3H, I**). The group with auxin levels below 3 mg·L^−1^ had a low induction rate but good rooting status (**Figures 3D–F, J**). However, the experimental group with high auxin levels (>3 mg·L^−1^) had a high induction rate but poor adventitious root quality, which was brittle, and the epidermis was swollen and loose (**Figure 3G, K, L**). In addition, the reduction in sucrose promoted root formation to a certain extent in the 1/2 MS basic medium but did not significantly increase the rooting percentage in the WPM basic medium.

### Acclimatization

Acclimatized *B. sinensis* plantlets exhibited a high survival rate of 72% after 6 weeks of acclimatization, and we observed the formation of a compound leaf from the simple leaf split. Plant material from the same batch of transplants grew consistently in the same medium and growth time; the adapted plantlets developed healthily and verdant green leaves, and exhibited typical morphological and growth traits of the plant species (**Figure 3C**).

### Genetic fidelity of regenerated shoots

We used 29 ISSR and 20 RAPD primers for the genetic fidelity evaluation. Of these, 91 distinct monomorphic bands were generated using five RAPD and seven ISSR primers, which were suitable for subsequent PCR amplification reactions (**Supplementary Table 1**). A single ISSR primer yielded an average of six bands for the ISSR markers, with banding patterns ranging from four (UBC-853) to eight (UBC-824) (**Supplementary Table 1**). Altogether, 44 bands were generated using ISSR primers, ranging in size from 250 to 3,000 bp (**Supplementary Table 1**). **Figures 4B, C** show the band patterns of UBC-874 and UBC-892, respectively. The RAPD primers yielded 47 bands with an average of 9 per primer. The average number of bands created by the isolated RAPD primer ranged from 6 (S226) to 13 (S208), and the bands ranged from 200 to 3,000 bp (**Supplementary Table 1**). The S130 and S144 banding patterns are shown in **Figures 4D, E**, respectively. The monomorphic banding patterns generated by DNA molecular markers demonstrate that the mother plants and regenerates have a stable genetic makeup. From the ISSR and RAPD markers, 44 and 47 monomorphic bands were generated, respectively.

## Discussion

Despite significant advancements in understanding *B. sinensis* through various genetic and ecological studies, practical conservation efforts are hindered by the species’ reproductive challenges. Current research on *B. sinensis* has mainly focused on chloroplast genomic resources, genetic divergence (Shang et al., 2022), demographic histories (Zhang et al., 2022), phylogeography, conservation genetics (Wang et al., 2018), and glucosinolate diversity (Montaut et al., 2015). Owing to the rare and endangered nature of *B. sinensis*, its unique pedigree, and geographical research significance, *ex situ* conservation and artificial expansion of this species have urgently necessitated further research. Our findings demonstrate a remarkable increase in germination rates, with over 50% of treated seeds sprouting after pulp removal—a significant improvement over natural germination rates observed in wild populations. Our study illuminates the physical dormancy mechanism inherent to *B. sinensis* seeds, where the seed coat and embedded chemicals impede germination, and showcases ayu novel approach to circumvent this barrier, enhancing germination success.

Owing to the lack of a well-established *in vitro* regeneration system, studies on the molecular mechanisms of regeneration in non-model woody plants are rarely reported, and a general belief stipulates that woody plants are more difficult than herbaceous plants for plant *in vitro* regeneration (Thorpe and Harry, 1990; Phillips and Garda, 2019; Abdalla et al., 2022; Nowakowska et al., 2022; Zdravković-Korać et al., 2022). Current research on the molecular mechanisms of plant *in vitro* regeneration is mainly focused on model plants, represented by *Arabidopsis thaliana* (Atta et al., 2009; Sugimoto et al., 2010; Zhai and Xu, 2021; Zhai et al., 2023). Many studies have shown that the use of PGRs exerts the most important effect on shoot proliferation during *in vitro* regeneration (El-Sayed et al., 2020; Fernandes et al., 2020). The cytokinin signaling pathway Arabidopsis histidine kinase-Histidine-containing phosphotransfer protein-Arabidopsis response regulator (AHK- AHP-ARR) activates the key gene WUSCHEL (WUS) for apical stem cell maintenance (Dai et al., 2017; Meng et al., 2017; Wang et al., 2017; Zhang et al., 2017), which through interaction with STM (Su et al., 2020), coordinately regulates the expression of CLV3. The WUSCHEL-SHOOT MERISTEMLESS-CLAVATA3 (WUS-STM-CLV) module ensures the morphogenesis of bud primordia. B-ARR transcription factors are key genes that activate the transformation of bud cell fate. On the one hand, B-ARR interacts with members of the CLASS III HOMEODOMAIN-LEUCINE ZIPPER (HD-ZIP III) family, directly binding to the *WUS* promoter to activate its transcription (Dai et al., 2017; Meng et al., 2017; Wang et al., 2017; Zhang et al., 2017), and on the other hand, it maintains the balance of endogenous auxin and cytokinin by inhibiting YUCCA (YUC)*1/4* (Cheng et al., 2013; Meng et al., 2017). Additionally, cytokinins and auxins influence the expression of the aforementioned genes by regulating histone modifications and the degree of chromatin openness (Wu et al., 2022). Auxin is also a decisive factor in initiating plant regeneration, with the auxin signaling pathway regulating cell division and pluripotency through key transcription factors, including members of the LATERAL ORGAN BOUNDARIES (LBD) family *LBD16/29* (Fan et al., 2012; Xu et al., 2018), GRAS family members SCR and SHR (Kim et al., 2018; Zhai et al., 2023), PLETHORA family members *PLT1/2* (Kareem et al., 2015; Zhai and Xu, 2021) and *PLT3/5/7* (Kareem et al., 2015; Varapparambath et al., 2022), and WUSCHEL-RELATED HOMEOBOX (WOX) family members *WOX11* (Liu et al., 2018) and *WOX5/7* (Kim et al., 2018; Zhai and Xu, 2021), all of which play significant roles in the establishment and maintenance of plant cell pluripotency. In plant *in vitro* regeneration, the ratio of exogenously added auxins and cytokinins influences the differentiation direction of regenerating seedlings. A regeneration study on *Andrographis echioides* demonstrated that the maximum regeneration efficiency and number of regenerations were achieved when the 6-BA/IAA ratio was 15/1 (Savitikadi et al., 2020). For *Populus trichocarpa*, a cytokinin/auxin ratio of 5/1 resulted in the highest bud induction rate (Ma et al., 2022). However, the specific ratios and intensities of these hormones have not been clearly determined across different plants, and varying these ratios with different hormone strengths can lead to dramatically different outcomes (Song et al., 2023). We observed both high and low induction rates at a 6-BA/NAA ratio of 3/1. The intensity of PGRs was also important; as the intensity of hormone combinations of the same ratio increased, shoot regeneration was first increased and then decreased; this phenomenon has also been reported during *in vitro* regeneration in other species (Shekhawat et al., 2021), indicating that both hormone combinations of ratio and intensity are important for the shoot regeneration rate (Yan et al., 2023). The *in vitro* regeneration of non-model plants typically relies on gradient hormone combinations or empirical experiments. Our findings pave the way for future investigations into the molecular mechanisms underpinning PGR effects on *in vitro* regeneration, crucial for refining micropropagation techniques across diverse plant species.

Elongation culture is a critical step in the *in vitro* regeneration system for *B. sinensis*, setting the stage for successful rooting by producing robust plantlets capable of resisting nutrient consumption by exogenous hormones. Our findings indicate that the choice of culture medium significantly impacts plantlet elongation and health, which formed different growth states and leaf colors: MS and 1/2MS media resulted in smaller adventitious shoots with curled, green leaves; WPM medium facilitated better shoot elongation and healthier green leaves, whereas DCR and 2× DCR media also supported shoot elongation but led to yellow and red leaf coloration, respectively. Studies on cultivated woody plants with obvious stems, such as *Eucalyptus benthamii* explant sources (Esposito-Polesi et al., 2020), *Larix olgensis* (Yan et al., 2023), Juniper species (Hazubska-Przybył, 2019), and *Persea americana* Mill. cv. Hass (Osorio et al., 2018), have achieved plantlet elongation by lowering the concentration of basic medium or the phytohormone concentration. In the present study, selecting the appropriate medium and reducing the nitrogen and phosphorus contents were conducive to adventitious shoot elongation at the stage of obvious stem differentiation (**Table 1**; **Figure 1**). A novel discovery in our study was the relationship between leaf coloration in *B. sinensis* and potassium ion concentration, underscoring the nuanced role of specific nutrients in plantlet development. This insight is critical for tailoring regeneration protocols to accommodate the unique physiological needs of woody plants. Woody plants have better-developed vascular tissues, and MS basic medium usually fails to yield good results in the gradual transition of large numbers of adventitious shoots to lignification. Previous anatomical studies have shown that the xylem vessel diameter of *B. sinensis* is small and water and inorganic salt transport is inefficient (Carlquist, 1996).

Rooting *in vitro* is a pivotal yet challenging step in the regeneration of woody plants, especially for recalcitrant and mycorrhizal species that exhibit unique rooting behaviors. Successful induction of rooting is essential for the regeneration of woody plants. Most of the recalcitrant species that are difficult to root are woody plants, which exhibit rooting differences. So far, many other plants are symbiotic with fungi in nature, which are difficult for tissue culture and *in vitro* regeneration system, and *B. sinensis*, which is a mycorrhizal plant, is one of them. For *in vitro* rooting, numerous studies favor the 1/2 MS basic medium and addition of auxin to induce rooting (Wei et al., 2017; Amiri et al., 2020). This study specifically aimed to optimize the *in vitro* rooting conditions for *B. sinensis* by comparing the efficacy of 1/2 MS and WPM media, which has lower inorganic salt concentration. A key finding of our research is that the 1/2 MS medium, with its balanced nutrient content, proved significantly more effective for *B. sinensis* rooting than the WPM medium. Our results underscore the critical balance between reducing inorganic salt concentration and ensuring sufficient nutrient availability for rooting, highlighting the inefficacy of solely reducing salts without considering overall nutrient needs. Normally, host plants translocate organic nutrients to mycorrhizal fungi, and mycorrhizal fungi translocate mineral nutrients to host plants (Wang et al., 2022). Under the aseptic conditions of *in vitro* regeneration, *B. sinensis* requires more nutrients for adventitious root formation, but no additional sucrose is required for root growth under aseptic conditions; therefore, the more nutrient-rich 1/2 MS with reduced sucrose would be more suitable for *in vitro* rooting of *B. sinensis* plantlets. The nuanced role of phytohormone concentrations in our study aligns with findings from research on other species, suggesting a general principle that overly high concentrations may hinder the development of a healthy root system. This insight is particularly valuable for the *in vitro* rooting of mycorrhizal woody plants. These results are consistent with those of research on red raspberries (*Rubus idaeus*) (Kim and Dai, 2020), which, despite the use of a high amount of auxin additive, does not necessarily result in good rooting conditions, as it makes the roots thick and loose, resulting in a fragile root system that is not conducive to rooting and transplanting. Similar conclusions have been reached for the cannon ball tree (*Couroupita guianensis* aubl.) (Shekhawat and Manokari, 2016).

Genetic fidelity studies play a crucial role in preserving the genetic integrity of plant species during the process of domestication and cultivation (Li et al., 2005). Nowadays, these studies become particularly relevant in plant tissue cultures, with the risk of somaclonal variations (Ferreira et al., 2023). This phenomenon, characterized by inconsistent traits in regenerated plantlets compared to the mother plant, presents challenges for large-scale production (Eeckhaut et al., 2020). Factors such as DNA methylation changes, oxidative stress, nutrient availability, and the use of high concentrations PGR have been identified as key contributors to these genetic and epigenetic alterations. For instance, studies on pineapple callus cultures and rice have highlighted the significant role of epigenetic modifications, such as DNA methylation changes, in driving somaclonal variations (Stroud et al., 2013; Lin et al., 2019). Moreover, oxidative stress (Bednarek and Orłowska, 2020), nutrient availability (Duarte-Aké and De-la-Peña, 2021), and the application of high PGR concentrations (Ferreira et al., 2023) may influence somaclonal variation, leading to genetic and epigenetic alterations in tissue culture plantlets. The development of stable and reliable regeneration systems becomes paramount, not only for mitigating the effects of somaclonal variation but also for enabling precise genetic studies and transformations. Such systems are essential for dissecting the genetic underpinnings of regeneration and mutation. Our protocol is controlled and reliable, as adventitious shoots transplanted more than 10 times are consistent with the genetic stability of first-generation donor material. Our findings align with similar genetic fidelity achievements in species like *Cornus alba*, *Thunbergia coccinea* Wall., *Manglietiastrum sinicum*, *Ranunculus wallichianus*, and *Haplophyllum tuberculatum* (Erişen et al., 2020; Srinivasan et al., 2021; Alsafran et al., 2022; Sultana et al., 2022; Luo et al., 2023), where direct organogenesis shoots exhibited consistent genetic profiles, as verified by ISSR and RAPD markers (Backiyarani et al., 2021).

One inherent limitation of our study stems from *B. sinensis*’s endangered status and small population, which restricted our sample size and, consequently, the scope of our preliminary research. The cherished and endangered status of *B. sinensis* prevents the performance of related studies. In the future, we aim to broaden our exploration of regeneration techniques by employing stems and leaves as explants, develop a comprehensive large-scale breeding system, and delve into the growth mechanisms of *B. sinensis*. The methodology established in this research advances the conservation of *B. sinensis* and sets a precedent for *ex situ* conservation and artificial propagation of other endangered species, providing a scalable model for biodiversity conservation, and this study also contributes valuable insights into the field of species-oriented plant tissue culture and provides a basic reference for further exploration of non-model woody plant regeneration.

## Conclusions

Our study presents the first fully effective regeneration system for *B. sinensis* seeds, marking a significant breakthrough in the species’ conservation efforts. We meticulously investigated the impact of PGRs at varying ratios and across different basic media, establishing a methodology that ensures successful large-scale production and conservation of *B. sinensis*. Utilizing ISSR and RAPD markers for clonal fidelity tests, we have demonstrated the genetic fidelity of the regenerated plants, further validating our protocol’s capacity to produce high-quality *B. sinensis* plantlets. Our efficient and reliable protocol transcends *B. sinensis* conservation, offering a promising model for the rescue and *ex situ* conservation of a wide range of endangered species. Our groundbreaking approach not only paves the way for the efficient *ex situ* conservation of *B. sinensis* but also sets a precedent for applying innovative regeneration techniques in the conservation of other endangered species, expanding the horizons of plant conservation research.

## Data availability statement

The original contributions presented in the study are included in the article/**Supplementary Files**. Further inquiries can be directed to the corresponding author.

## Author contributions

XY: Data curation, Investigation, Methodology, Validation, Visualization, Writing – original draft, Writing – review & editing. KZ: Conceptualization, Data curation, Investigation, Methodology, Validation, Writing – original draft. PL: Resources, Validation, Writing – review & editing. XZ: Validation, Writing – review & editing. ZZ: Investigation, Resources, Validation, Writing – review & editing. HZ: Formal analysis, Methodology, Resources, Validation, Writing – review & editing. MZ: Conceptualization, Funding acquisition, Investigation, Project administration, Supervision, Writing – review & editing.
